# Foraging history of individual elephants using DNA metabarcoding

**DOI:** 10.1098/rsos.230337

**Published:** 2023-07-05

**Authors:** Brian A. Gill, George Wittemyer, Thure E. Cerling, Paul M. Musili, Tyler R. Kartzinel

**Affiliations:** ^1^ Department of Ecology, Evolution, and Organismal Biology, Brown University, Providence, RI 02912, USA; ^2^ Institute at Brown for Environment and Society, Brown University, Providence, RI 02912, USA; ^3^ Department of Fish, Wildlife, and Conservation Biology, Colorado State University, Fort Collins, CO 80523, USA; ^4^ Save the Elephants, Nairobi, Kenya; ^5^ Department of Geology and Geophysics, University of Utah, Salt Lake City, UT 84112, USA; ^6^ Department of Biology, University of Utah, Salt Lake City, UT 84112, USA; ^7^ Botany Department, East African Herbarium, National Museums of Kenya, Nairobi, Kenya

**Keywords:** forage maturation hypothesis, grazer–browser continuum, megaherbivore, niche variation hypothesis, optimal foraging theory, social foraging

## Abstract

Individual animals should adjust diets according to food availability. We used DNA metabarcoding to construct individual-level dietary timeseries for elephants from two family groups in Kenya varying in habitat use, social position and reproductive status. We detected at least 367 dietary plant taxa, with up to 137 unique plant sequences in one fecal sample. Results matched well-established trends: elephants tended to eat more grass when it rained and other plants when dry. Nested within these switches from ‘grazing’ to ‘browsing’ strategies, dietary DNA revealed seasonal shifts in food richness, composition and overlap between individuals. Elephants of both families converged on relatively cohesive diets in dry seasons but varied in their maintenance of cohesion during wet seasons. Dietary cohesion throughout the timeseries of the subdominant ‘Artists’ family was stronger and more consistently positive compared to the dominant ‘Royals’ family. The greater degree of individuality within the dominant family's timeseries could reflect more divergent nutritional requirements associated with calf dependency and/or priority access to preferred habitats. Whereas theory predicts that individuals should specialize on different foods under resource scarcity, our data suggest family bonds may promote cohesion and foster the emergence of diverse feeding cultures reflecting links between social behaviour and nutrition.

## 1. Introduction

Dietary differences between individuals of the same species are common in nature, with broad implications for animal behaviour, community dynamics and evolution [1,2]. Individual dietary differences underpin the population's total niche width [3–5], which can vary as individuals' diets become more or less similar to each other when they shift, expand or contract [6,7]. These patterns of change are governed by a balance between intraspecific competition that increases among-individual variation versus interspecific competition that constrains a population's total niche width [8–10]. In social animals, group foraging can theoretically confer advantages over solitary foraging when it enables individuals to locate high-quality foods [11]. Social foraging may foster dietary cohesion within groups, but individuals that forage together may often still obtain different diets if they vary in dominance, age, reproductive state or other characteristics that alter their nutritional needs, preferences or access to resources [12,13]. The growing awareness that individual diets differ compels us to understand individual-level dietary variation [14], though fine-grained timeseries on individuals' diets remain sparse and difficult to obtain.

As the largest terrestrial herbivore, elephants’ mass and hindgut fermentation digestive system require bulk foraging behaviour, but they often target high-value foods when available [15,16]. They tend to dominate terrestrial resources over other species [17], but intraspecific competition is strong and generally structures space use and resource access [18]. Elephants take advantage of diverse resources as reflected in the breadth of their diets: they eat divergent plant growth forms (e.g. herbs, trees, succulents), parts (leaves, fruits, bark, twigs) and lineages (e.g. monocots, eudicots) [15,19,20]; they select for high-quality plant products (e.g. fruits), including human agricultural resources [21], as well as prioritize access to peak forage biomass over peak forage quality (i.e. where plants ‘brown down’) [22]; they even scavenge refuse from garbage dumps where overall food quantity can provide better nutrition than natural forage [23]. Reflecting this versatility, individual elephants have been shown to employ a diversity of resource-selection tactics when foraging in the same environment [24], which in turn control the diversity and abundance of plant species across their range [16,25]. An open question concerns the extent of individual-level dietary variation and the potential for intraspecific competition among elephants that forage together.

The elephant population in the semi-arid habitats of Samburu and Buffalo Springs National Reserves, Kenya has been monitored since 1997 [24,26,27]. Detailed observations of two moderately sized families—the ‘Royals’ and ‘Artists’ that each comprised a core of 4–5 adult females—enable comparisons of individual-level movements and foraging histories. Core members of each family travel as a cohesive unit, making use of habitats throughout both reserves and constricting their ranges to the proximity of the Ewaso N'giro River to access riparian vegetation in dry seasons [28]. In previous studies, timeseries of carbon stable isotopes in elephant hairs from these families revealed similar seasonal increases in their proportional consumption of C_4_ plants (predominantly grasses) versus all other C_3_ plants when grass availability rose following rains [27,28]. Yet despite ostensibly similar seasonal patterns of grass consumption, members of the two families may have differed in their use of the greater than 200 C_3_ and C_4_ plant species that occur patchily throughout the landscape and across seasons. Indeed, long-term studies have shown that the Royals have higher social ranks and priority access to preferred foraging areas over the Artists [18,29]. For all of these reasons, members of each family group may exhibit cryptic differences in social foraging strategies despite their ostensibly similar responses to seasonal changes in the abundance and diversity of plant species [30].

Two theoretical frameworks provide contrasting expectations about the influences of individualistic and cohesive group-level foraging behaviors in elephants: Optimal Foraging Theory [31–33] and the Niche Variation Hypothesis [34]. Both frameworks predict group-level diets should expand to mitigate intraspecific competition when resources are limited and contract around a subset of preferred resources when they are plentiful, but they diverge in the mechanisms by which diet expansions should occur [35]. Under Optimal Foraging, individuals cohesively expand diets when they are all forced to become accepting of otherwise undesirable resources [31–33]. In contrast, the Niche Variation Hypothesis posits that group members will individualistically specialize on different subsets of limited resources in ways that ameliorate intraspecific competition [1,4]. Clearly, elephant foraging strategies should vary with the abundance, diversity and desirability of accessible foods—but are there consistent seasonal patterns to the influence of individualistic versus cohesive foraging behaviors?

There are at least three plausible seasonal patterns in elephant foraging strategies that could shift the balance of individualistic versus cohesive influences on group behaviour—these patterns may or may not be consistent among individuals. First, and in accordance with both Optimal Foraging and the Niche Variation Hypothesis, dietary diversity and differentiation may be greatest during dry periods when previously avoided resources become acceptable and preferred resources become scarce: staple plants favoured by elephants may lose their leaves in the dry season such that elephants must incorporate species with more drought-tolerant leaves out of necessity [36]; the ratio of crude protein to plant secondary metabolites can make some plant species more palatable as defence chemistry weakens in the dry season [37]; adherence to the Detoxification Limitation Hypothesis may require elephants to employ a dietary mixing strategy to avoid overwhelming their detoxification systems by feeding continually on the few plant species that can meet their energetic demands [38,39]. Second, and opposing these expectations, elephants could increase their dietary richness in wet seasons because this is when the most plant species are available and vegetation quality is generally highest, obviating the need to feed selectively [40]. Third, environmental changes may generate non-monotonic seasonal trends: intermediate levels of resource availability can correspond to times of either ‘green-up’, when individuals may concentrate their feeding on a subset of plant species that produce flushes of high-quality forage early in a wet season [41], or ‘brown-down’, when individuals may prioritize high-biomass energy sources over protein-rich foods [22]. Variation in elephant diets could thus be maximized: (i) in wet seasons when individuals select from the greatest abundance and diversity of plant species; (ii) in dry seasons when intraspecific competition for a limited subset of plant species prevents selective foraging; or (iii) without a monotonic trend if both seasonal extremes—or the transitions between them—evoke different nutritional or social foraging priorities [20]. Determining whether and how elephants from the same family groups seasonally partition their use of plant diversity could help clarify how social foraging behaviours are structured in relation to seasonal variation in food availability [18,42].

We used fecal DNA metabarcoding to construct dietary timeseries for the four core adult elephants in the Artists family over approximately four months and two core adults in the Royals family over approximately 14 months that included seasonal pulses in vegetation greenness. We coupled analyses of carbon stable isotopes from feces and hair with dietary DNA metabarcoding, GPS-tracking and remote-sensing data to evaluate individual diet variation. We hypothesized that: (i) dietary richness would increase and composition would be maximized during seasonal pulses of greenness, when individuals can access more plant species and a particularly species-rich array of grasses and (ii) foraging strategies rooted in strong social bonds and the shared need to maximize food intake would produce generally strong dietary cohesion within family groups, with an uptick in the degree of foraging individuality associated with resource diversity in the wet season. With respect to hypothesis (ii), we were interested in how social foraging ecology governs individual-level elephant foraging behaviors based on variation in their degree of social dominance and responsibility for rearing young calves [43].

## 2. Methods

### 2.1. Study system

**Figure 1.**
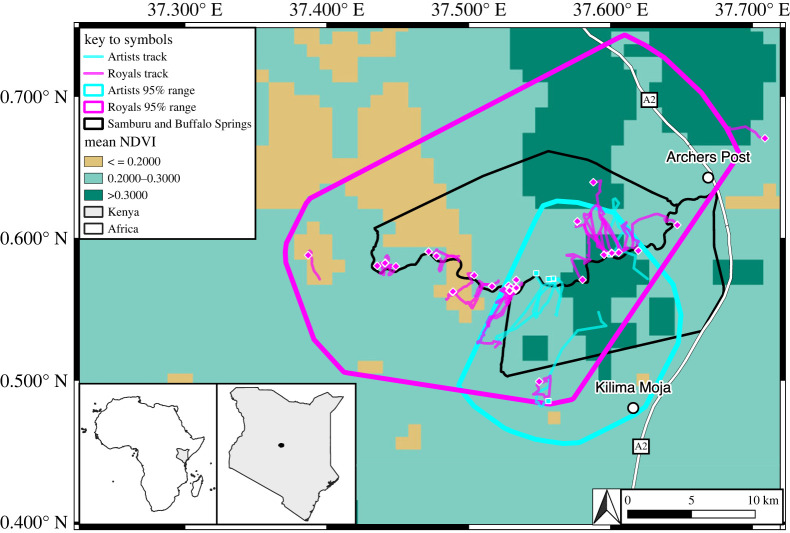
Samburu and Buffalo Springs National Reserves. The polygons map 95% ranges for Artists (blue) and Royals (pink) during the two timeseries. Diamonds show the start of tracks at 12:00 PM the day prior to sampling and squares show endpoints at 12:00 PM the day of sampling. The background shows mean NDVI over both study periods (mean of 21 images for 1-Jul-2001 to 21-Jan-2002 and 51 images for 1-Jul-2005 to 21-Nov-2006). The Ewaso N'giro river runs between the boundaries of Samburu and Buffalo Springs.

Our sampling strategy focused on establishing timeseries of dietary variation within and among core members of the Artists and Royals elephant families at Samburu and Buffalo Springs National Reserves, which are protected areas that meet at the Ewaso N'giro River in Kenya (figure 1) [26]. The habitat is characterized by riverine woodland dominated by river acacia (*Vachellia elatior*) and palms (*Hyphaene coriacea*) as well as low-lying pans with saline soil dominated by shrubs (*Salsola dendroides*), scrub woodland, and wooded grassland [26,44]. There are pronounced seasonal pulses of rainfall punctuated by extended dry periods, with mean annual rainfall of approximately 350 mm (1957–2016) that generally accumulates between March–May and October–December [18].

We obtained a total of 69 elephant fecal samples across two representative timeseries, each representing the core adult members of one family. The core adult members of each elephant family are observed together in 85–100% of sitings [45], so they have access to virtually identical resources and GPS telemetry can be used to quantify group-level habitat use. At the time of study, the Artists' core family group comprised four adult females, and we obtained overlapping timeseries data from 100% of them. This timeseries included 20 samples from the four Artists from August–December 2001: five samples each from Goya (♀; 41 years old), Flaubert (♀; 12 years old), Rodin (♀; 25 years old) and Matisse (♀; 21 years old). The Royals’ core family group comprised five adult females, and we obtained relatively lengthy and high-density timeseries for two of them (40%). This timeseries included 49 samples from two Royals from August 2005–October 2006: 25 samples from Anastasia (♀; 32 years old) and 24 samples from Cleopatra (♀; 40 years old; electronic supplementary material, dataset S1). During the times of sampling, calf dependency varied both within and among groups. No member of the Artists core group was pregnant, though three had calves (Goya, Flaubert and Rodin), the youngest of which was greater than 2 years old. All Artist calves were not lactationally dependent. For the Royals, by contrast, both individuals were pregnant at the start of sampling and Cleopatra gave birth shortly after sampling began (13 Oct 2005); the next youngest calves ranged from approximately 2 to 3 years old for Anastasia and Cleopatra, respectively. Fresh samples were collected over areas of 113 and 336 km^2^ for Artists and Royals (figure 1), respectively. Samples were dried and stored at room temperature for 14–19 years until split for dietary DNA and carbon stable isotopes analyses based on stringent quality control procedures described in electronic supplementary material, appendices S3–S5, and summarized below.

### 2.2. Elephant tracking

We used GPS collars to (i) calculate 95% home ranges of each family during the study periods, (ii) calculate overlap of the two families' 95% home ranges and (iii) track Goya's (representing Artists) and Anastasia's (representing Royals) habitat usage prior to sample collection (electronic supplementary material, dataset S2). We used satellite-based remote sensing to calculate normalized difference vegetation index (NDVI) across the habitats that elephants used during each study period, as this metric provides a useful indicator of food quality and availability for elephants with good spatial and temporal coverage in the system [28,46]. Precipitation-driven pulses of food quality and availability generated significant correlations between 10-day cumulative rainfall and mean 10-day composite NDVI in both timeseries (electronic supplementary material, datasets S3 and S4, appendix S1). Although estimates of elephant gut passage times can range from less than 1 day to more than 4 days, depending on the material composition and intake rate, most material passes in 30–40 h [47–49]. We thus assumed fecal samples represent approximately 24–48 h of foraging activity and used the remote-sensing data to estimate the mean NDVI and mean rate of change in NDVI (ΔNDVI) to represent habitats occupied by elephants over 24 h prior to sampling (electronic supplementary material, dataset S1, appendix S1).

### 2.3. Carbon stable isotopes

We measured the ratio of ^13^C/^12^C (*δ*^13^C) to estimate % C_4_ plants in diets. We used an isotope ratio mass spectrometer following combustion on a flow-through modified Carlo-Erba system, with values in permil (‰): $\delta^{13}C=(({(}^{13}C{/}^{12}{C)}_{\mathrm{sample}}/{(}^{13}C{/}^{12}{C)}_{\mathrm{standard}})-1)\times 1000$. We used the Vienna PeeDee Belemnite standard. We converted $\delta^{13}C$ values to % C_4_ using a Bayesian mixing model in *simmr* [50] (electronic supplementary material, dataset S1). For C_3_ plants, we used a source end-member value of −27.43‰ ± 0.98 based on measurements of 173 local plant samples (electronic supplementary material, dataset S5). For C_4_ plants, we used a source end-member value of −13.37‰ ± 0.95 based on measurements of 80 local plant samples (electronic supplementary material, dataset S5). For both C_3_ and C_4_ plants we used a correction value of −0.77 ± 0.34 to account for $\delta^{13}C$ depletion of feces compared to dietary inputs [51]. Before ecological analyses, we conducted a sensitivity analysis to assess the reliability of our protocols for quantifying fecal *δ*^13^C (electronic supplementary material, appendix S2, figure S1).

### 2.4. Dietary DNA analysis

We coupled fecal DNA metabarcoding with an extensive plant DNA barcode library from the region to identify dietary plants (electronic supplementary material, appendices S3–S5). Briefly, we extracted DNA, used PCR to amplify the P6 loop of the chloroplast *trn*L (UAA) intron [52], and conducted paired-end sequencing on an Illumina MiSeq. We selected the *trn*L-P6 marker because it is highly variable among plant species despite its relatively short length (mode ≈52 base pairs per species), which enables relatively precise identification of food plant species even when samples contain relatively degraded DNA fragments [52], and because a regional DNA barcode library developed from an extensive collection of herbarium-vouchered plant specimens is available to facilitate taxonomic identifications [19,53,54]. We first aligned forward and reverse sequence reads, tallied identical sequences, and filtered putative sequencing errors using OBITOOLS [55] (electronic supplementary material, appendix S4). We then used OBITOOLS to identify sequences based on similarity to reference sequences from each of two plant DNA barcode libraries: (i) the near-comprehensive plant DNA barcode library that was developed for the region at Mpala Research Centre, Kenya, which comprised 312 unique sequences (i.e. a ‘local’ library) [53] and (ii) the European Nucleotide Archive v. 141, which comprised 22 385 unique sequences (i.e. a ‘global’ library) [40]. Prior to analysis, we further filtered the results of putative errors by requiring dietary DNA sequences to exactly match a reference sequence in one of these reference libraries (electronic supplementary material, appendix S5) [56]. When inferring plant taxonomy, we gave preference to the local library, but accepted identifications based on the global library when a sequence was not present in the local library (electronic supplementary material, dataset S6, appendices S4–S5). We dropped one fecal sample that yielded too few DNA reads (*n* = 166) and rarefied the dataset to the minimum sequence depth of the 68 remaining samples (13 490 reads; electronic supplementary material, dataset S7) before calculating the DNA sequence relative read abundance (RRA) of each taxon in each sample (electronic supplementary material, dataset S8). Estimating proportional consumption of plant species has been controversial, but computer simulations and experimental data show that RRA is often reliable [57,58] and that alternative strategies based on presence/absence data can be more prone to error [56,59]. We further assessed the reliability of our bioinformatic pipelines for assembling and filtering sequence data as well as identifying dietary taxa using the two plant DNA barcode libraries based on extensive sensitivity analyses (electronic supplementary material, appendices S4–S5, figure S2). We then calculated dietary richness and diversity using Hill numbers to up- or down-weight the influence of rare species on seasonal trends in diversity (i.e. Hill numbers with *q* = 0 and 1, respectively) using *hillR* [60].

### 2.5. Statistical analyses

We expected that there would be a positive correlation between % grass DNA in fecal samples and the corresponding % C_4_ consumption as inferred using carbon stable isotopes, indicating the methods provide similar estimates of consumption. For each elephant family separately, we used linear regression to test if isotopic % C_4_ could be used to predict % grass RRA.

We used three approaches to test for significant seasonal differences in dietary richness and composition for each elephant family separately (hypothesis (i)). First, we tested whether dietary richness increased significantly with NDVI using stepwise polynomial regression. We modelled dietary richness as linear, quadratic and cubic polynomials of NDVI, testing for significant improvements in model fit with the addition of additional terms until there was no appreciable gain using ANOVA F-tests. Second, we tested for significant differences in diet composition using Bray–Curtis dissimilarity and permutational multivariate analysis of variance (perMANOVA) in *vegan* [61]. The perMANOVAs included the predictors NDVI, ΔNDVI, and the NDVI × ΔNDVI interaction. Third, to identify plant taxa that contributed strongly to seasonal dietary differences, we modelled differential representation of (i) each DNA sequence and (ii) all DNA sequences representing each plant family as a function of NDVI using DESeq2 [62]. DESeq2 uses raw sequence counts to evaluate the strength and significance of changes in response to conditions.

**Figure 2.**
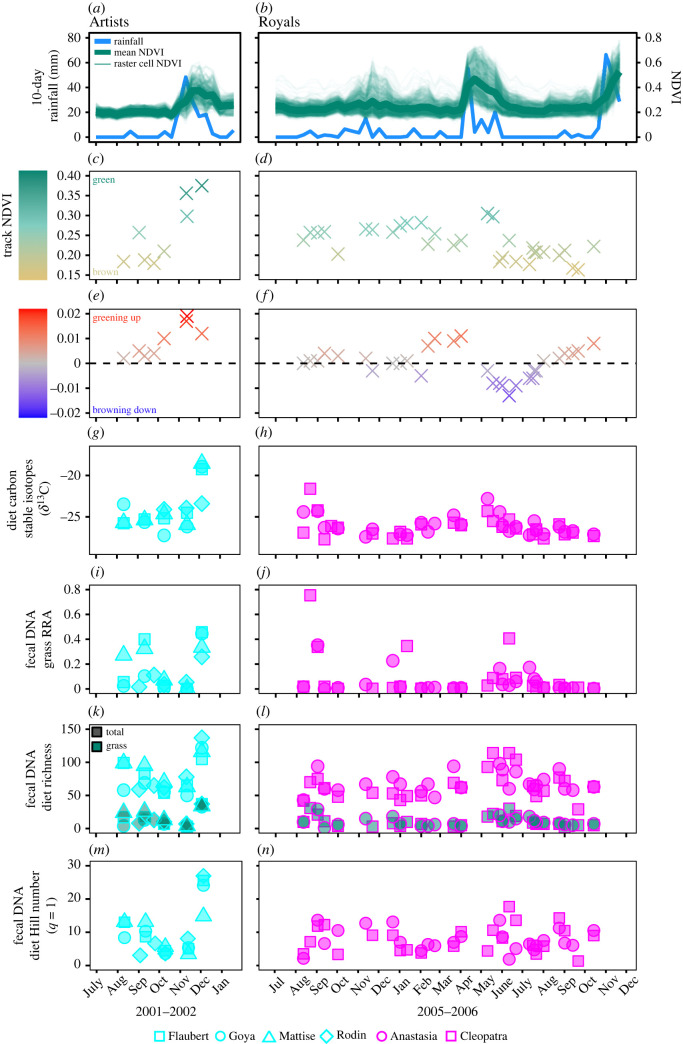
Seasonal trends in elephant environments and diets. Timeseries represent fecal samples from six elephants (Artists, blue; Royals, pink). (*a,b*) The cumulative sum of rainfall over 10 days prior to NDVI measurements is shown together with lines representing (i) NDVI in raster cells traversed by elephants (light green) and (ii) mean NDVI across each home range (dark green). These data were used to differentiate seasons (Artists: dry = Aug–Oct 2001, wet = Nov–Dec 2001; Royals: wet = Nov 2005 and Apr–Jun 2006, dry = Aug–Oct 2005, Dec 2005, Jan–Mar 2006, Jul–Oct 2006). The mean (*c,d*) NDVI and (*e,f*) ΔNDVI of raster cells traversed were calculated for the day of sampling. Fecal carbon stable isotopes revealed seasonal variation in (*g,h*) *δ*^13^C in diets. Fecal DNA revealed seasonal variation in (*i,j*) % grass RRA, (*k,l*) dietary species richness (overall and for the subset of grass species), and (*m,n*) dietary diversity.

For each timeseries separately, we also tested for significant familial cohesion and individual constancy in diet (hypothesis (ii)). We partitioned dietary variation within and between individuals' timeseries based on discrete wet and dry periods that reflected NDVI (figure 2). We began by calculating grand means of Bray–Curtis dissimilarity between all samples from each timeseries, which represented baseline (null) levels of dietary variation. We then calculated mean dietary dissimilarities (i) between individuals and (ii) within the same individual for each season. Since there were six pairwise comparisons involving Artist individuals, we calculated dietary dissimilarities for each pair separately. We tested whether these dissimilarities differed significantly from the grand means by permuting each dietary dissimilarity matrix 999 times and calculating 2-tailed *p* values. Positive and significant results indicate samples are more similar than expected based on random draws from the timeseries (i.e. indicative of familial cohesion or individual constancy); conversely, negative values indicate diets are significantly more dissimilar than random (i.e. individual specialization or individual variability). A sensitivity analysis to determine whether including all dietary taxa that passed filtering versus excluding ‘rare’ taxa (below a 1% threshold) in these analyses would produce different results revealed no qualitative difference in the seasonal trends (electronic supplementary material, appendix S5).

## 3. Results

### 3.1. Foraging histories

We sampled both elephant families in dry and wet seasons (figure 2). Artists were sampled through a unimodal green up in 2001 (single season) whereas Royals were sampled for a full year covering two seasonal pulses (smaller in November 2005; larger in April 2006; figure 2). The median distance moved over 24 h prior to sampling by the Artists was 9 km (range = 4–17 km) and by the Royals was 4 km (range = 2–9 km; electronic supplementary material, dataset S1). Owing in part to the shorter duration of the Artists' timeseries, our data reflect the foraging history of the Artists over a smaller spatial extent (Artists 95% home range size = 223 km^2^ during sampling; Royals 95% home range size = 632 km^2^ during sampling; spatial overlap of home ranges = 148 km^2^; figure 1). The NDVI of areas traversed over 24 h prior to sampling was comparable between the Artists (median = 0.21; range = 0.18–0.38) and Royals (median = 0.23; range = 0.16–0.31; figure 2; electronic supplementary material, dataset S1). Values of ΔNDVI for the Artists were all positive and generally increased from August to December 2001, whereas they varied between positive and negative for the Royals given that sampling spanned multiple seasonal pulses during this longer sampling period (figure 2).

### 3.2. Carbon stable isotopes

The Artists’ diets produced median fecal *δ*^13^C values of −24.73 (range = −27.06 to −18.36) and the Royals' had a median of −26.33 (range = −27.73 to −21.63), both reflecting relatively low overall % C_4_ values that increased with NDVI (Artists median = 20% C_4_ consumption, range = 8–65%; Royals median = 11%, range = 6–41%; figure 2; electronic supplementary material, dataset S1).

### 3.3. Dietary DNA

**Figure 3.**
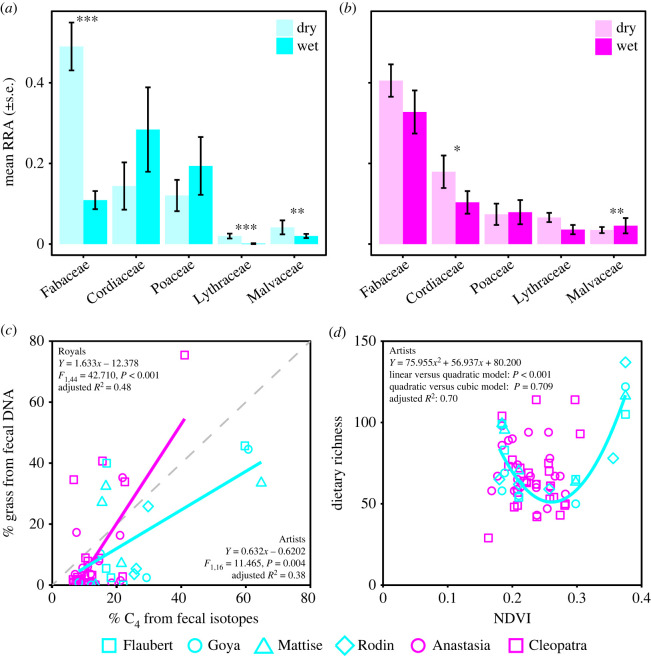
DNA metabarcoding revealed seasonal dietary changes. (*a*) Artists (blue) and (*b*) Royals (pink) exhibited differential consumption of plant families by season. The top-5 plant families in descending order of mean RRA are shown, with significant differences determined using NDVI in DESeq2 (accounting for multiple comparisons; see electronic supplementary material, datasets S10–S13, for all results). (*c*) A correlation between % C_4_ using fecal isotopes and % grass DNA differed between timeseries families (the dashed grey line is the 1:1 line). (*d*) Quadratic polynomials best approximated the relationship between dietary richness and NDVI overall (results in figure) and for the Artists' timeseries (*Y* = 75.955*X*^2^ + 56.937*X* + 80.200, linear versus quadratic model: *p* < 0.001, quadratic versus cubic model: *p* = 0.709, adjusted *R*^2^ = 0.70). There was no significant correlation between dietary richness and NDVI for Royals (all *p* > 0.05).

Dietary DNA yielded 5 755 900 sequence reads, including 3 samples sequenced two times and 5 samples sequenced three times (median = 72 374 reads per sample; range = 166–128 879; electronic supplementary material, dataset S6). After filtering and rarefying the data (electronic supplementary material, dataset S7), calculating RRA (electronic supplementary material, dataset S8), and eliminating replicate samples (electronic supplementary material, dataset S9), our final dataset comprised 68 samples and represented 62 plant families, 167 genera and 367 unique *trn*L-P6 sequences (151 of which matched a single named plant species and all of which were identified to family level using the local and global reference libraries; electronic supplementary material, datasets S1 and S9). The five plant families with the greatest mean RRA across samples included Fabaceae (acacias and other legumes), Cordiaceae (e.g. *Cordia*), Poaceae (grasses), Lythraceae, and Malvaceae (e.g. *Grewia*), which cumulatively accounted for a mean of 73% and 71% of diets in the Artists' and Royals’ timeseries, respectively (figure 3). As many as 137 plant taxa occurred in a single dietary sample (Rodin on 5 December 2001), with medians of 70 taxa per sample for Artists (range = 50–137) and 64 for the Royals (range = 29–114; electronic supplementary material, dataset S1). Diet diversity distributions were highly skewed and dominated by a relatively small number of food taxa, resulting in a much lower effective number of species per sample when using Hill numbers to down-weight the influence of rare taxa (i.e. ‘diversity’ was always less than 30 effective species per sample; Artists' median = 8; Royals’ median = 7; figure 2). Seasonal trends in total richness and the effective number of species were similar through both timeseries, as elephants foraged predominantly on a subset of available resources through time (figure 2*k–n*). Accordingly, only 20 plant taxa represented ≥1% RRA of across all samples and the mean RRA of these sequences was 85% per sample (Artists: 77%; Royals: 88%); only 3 taxa achieved ≥5% RRA across all samples and these accounted for a mean of 49% RRA per sample (Artists: 46%; Royals: 50%). Given that the samples had been stored at room temperature for 14–19 years, the data reflect remarkable plant species diversity. The vast majority were expected components of elephant diets, although very low levels of DNA from taxa not known to occur in the study area could reflect contamination during storage (e.g. Pinaceae, ‘present’ in 6 samples: mean RRA = 0.04%; *Quercus*, ‘present’ in 8 samples: mean RRA = 0.44%; electronic supplementary material, dataset S9).

### 3.4. Statistical analyses

For both timeseries separately, % grass DNA was positively correlated with % C_4_ estimated from stable isotopes (figure 3). Median grass consumption was ≤20% along both axes, so these comparisons were anchored by low values near the 1:1 line (figure 3). Because the Royals' timeseries included denser sampling over a longer period, we obtained many samples that fell near the origin of the plot for this timeseries (i.e. low % grass DNA and % C_4_ in figure 3). Although dung and hair tissues incorporate isotopes over different timescales and occasionally diverge (e.g. if the sampled dung bolus is not representative of all foods consumed that week; electronic supplementary material, figure S3), we found the isotopic estimates of % C_4_ from dung and hair chronologies were strongly correlated for the Artists and marginally significantly correlated for the Royals (electronic supplementary material, figure S4). These correlations are notable given the different timespans the samples represent: fecal samples contain material representing approximately 1–2 days of foraging activity collected every few weeks, whereas the isotopes in tail hairs represent approximately 7 days of activity. Yet the temporally fine-grained fecal data did not reveal a clear and consistent seasonal ‘switch’ between grass- and non-grass-based diets to match expectations. Only the Artists' timeseries revealed a positive and significant correlation between fecal isotopes (*δ*^13^C and % C_4_) and the 10-day composite NDVI values; % grass DNA was not significantly correlated with NDVI in either timeseries (electronic supplementary material, figure S5). There was a slight increase in mean % grass DNA between dry and wet seasons, but the result was not significant after correcting for multiple comparisons involving many plant families (figure 3; electronic supplementary material, datasets S10 and S11). A noteworthy similarity between the two timeseries is that the plant families with the greatest relative abundance tended to be the staples of dry season diets and became less concentrated following the diet expansions of wet seasons (e.g. Fabaceae), but there was otherwise little correspondence between the timeseries (figure 3; electronic supplementary material, figure S6).

**Figure 4.**
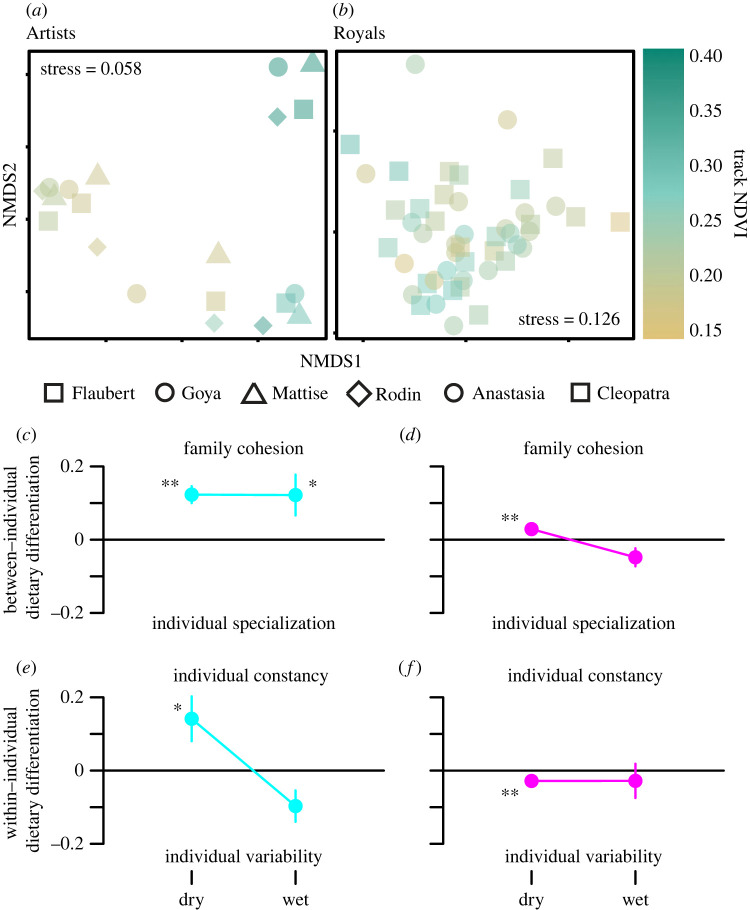
Within- and between-individual diet variation. A non-metric multidimensional scaling (NMDS) was calculated separately for each timeseries for (*a*) Artists and (*b*) Royals. Point colours represent NDVI (see electronic supplementary material, figure S7, for identical plots colorized by ΔNDVI). Comparisons reveal (*c,d*) between- and (*e,f*) within-individual variation for elephant families across seasons (Artists, blue; Royals, pink). Mean differences from each timeseries’ grand mean Bray–Curtis dietary dissimilarity are plotted ± 1 s.e. Significant positive values represent (*c,d*) familial cohesion or individual constancy (*e,f*) while negative values represent (*c,d*) individual specialization or (*e,f*) individual variability. Significant differences from zero, which represents the null expectation provided by the grand mean of dietary dissimilarity, are indicated as 2-sided *p* values (*p < 0.05, **p < 0.01). Exact Bray–Curtis values for the pairwise comparisons are shown as a timeseries in electronic supplementary material, figure S8. Separate pairwise comparisons for all Artists are shown in electronic supplementary material, figure S9. A sensitivity analysis testing the robustness of these results to the inclusion of rare dietary DNA sequences is shown in electronic supplementary material, figure S10.

We did not find a monotonic increase in dietary richness with NDVI (contrary to hypothesis (i)), but there were significant seasonal differences in diet composition. The Artists’ timeseries revealed significant increases in dietary richness at seasonally low and high NDVI extremes (described by a quadratic polynomial in figure 3). In contrast, NDVI was not a significant predictor of dietary richness in the Royals' timeseries. Artists exhibited relatively strong and significant differences in diet composition based on NDVI (perMANOVA, pseudo-*F*_1,16_ = 9.5, *R*^2^ = 0.29, *p* ≤ 0.001), ΔNDVI (pseudo-*F*_1,16_ = 2.7, *R*^2^ = 0.08, *p* = 0.025), and the NDVI × ΔNDVI interaction (pseudo-*F*_1,16_ = 4.4, *R*^2^ = 0.13, *p* = 0.003; figure 4; electronic supplementary material, figure S7). With increasing NDVI, the Artists significantly increased relative consumption of 34 plant taxa and reduced consumption of 12 (all adjusted *p* < 0.05; electronic supplementary material, dataset S12). By contrast, the Royals’ timeseries revealed only weak, albeit significant, dietary differences based on NDVI (perMANOVA, pseudo-*F*_1,44_ = 2.4, *R*^2^ = 0.05, *p* = 0.008) and ΔNDVI (pseudo-*F*_1,44_ = 2.2, *R*^2^ = 0.04, *p* = 0.016) with no significant NDVI × ΔNDVI interaction (pseudo-*F*_1,44_ = 1.4, *R*^2^ = 0.03, *p* = 0.160; figure 4; electronic supplementary material, figure S7). Accordingly, there was no significant differential use of plant taxa as a function of NDVI for the Royals' timeseries (electronic supplementary material, dataset S13).

Hypothesis (ii), that family bonds generally foster dietary cohesion, was supported for both timeseries in dry seasons but the expected uptick in wet-season individuality was only seen in the Royals’ timeseries (figure 4). Grand mean Bray–Curtis values were 0.72 and 0.67 for Artists and Royals, respectively. These values reflect variation both within individuals (mean for Artists = 0.78; Royals = 0.67) and between individuals of the same core family group (Artists = 0.71; Royals = 0.67), whereby time-matched comparisons of diet composition revealed greater overlap between individuals at the same point in time than within individuals at different points through time (time-matched dissimilarity values interquartile ranges for Artists' and Royals’ timeseries: 0.35–0.50 and 0.42–0.63, respectively; electronic supplementary material, figure S8). The four Artists exhibited strong and significant dietary cohesion in both dry (*p* ≤ 0.001) and wet seasons (*p* = 0.014), with high individual constancy in the dry season (*p* = 0.027) that declined in the wet season (*p* = 0.562; figure 4; electronic supplementary material, figures S8 and S9). In contrast, the two Royals had weak, but significant, dietary cohesion in the dry season (*p* = 0.004) that declined substantially toward a marginally significant degree of individual specialization in the wet season (*p* = 0.059; figure 4; electronic supplementary material, figures S8 and S9); sensitivity analyses reinforced this result because the apparent degree of individual specialization by Royals in the wet season became stronger and statistically significant when focusing only on the subset of relatively dominant foods and excluding rare taxa from consideration (electronic supplementary material, appendix S5, figure S10). The two Royals also showed a significant degree of individual variability in the dry season (*p* = 0.008) that changed little but was not significant in the wet season (*p* = 0.452; figure 4; electronic supplementary material, figures S8 and S9). This contrast is especially notable given the larger number of Artists that we tracked (*n* = 4; 100% of core adult females) compared to Royals (*n* = 2; 40% of core adult females). Close inspection of all pairwise comparisons involving the four Artists revealed that Matisse, the only core member of the family without a calf at the time of sampling, tended to exhibit low cohesion (high dissimilarity) except at the onset of the rainy season (electronic supplementary material, figures S8 and S9).

## 4. Discussion

We revisited a model system on the foraging ecology of individual animals using an archive of fecal samples that enabled us to reconstruct taxonomically precise dietary timeseries for two elephant families. Dietary stable isotopes reaffirmed a well-established pattern whereby elephants increased proportional consumption of C_4_ plants following rainfall-driven pulses in NDVI [27,28,46] and dietary DNA revealed fine-grained differences in the proportional consumption of plant species eaten (figures 2 and 3). For both timeseries, grasses constituted only a relatively small fraction of total dietary richness such that non-grasses cumulatively accounted for ≥80% of diets and contributed strongly to the seasonal patterns. Elephants incorporated a diversity of new C_3_ and C_4_ plant species into their diets with pulses of productivity, and some (but not all) of them also expanded their diets during extended dry periods, such that diets could be observed to shift quickly following ‘green-up’ and become most concentrated on a narrow subset of regional plant diversity during seasonal transitions.

### 4.1. Cohesion and individuality

Dietary DNA varied considerably within and among elephants in both families. Because these elephants centre their distributions on riparian habitats during dry seasons and travel further in wet seasons [28], we expected diets to reflect this spatial behaviour. As predicted, the individuals sampled from both family groups diversified their diets following seasonal pulses in rainfall (figure 2) and exhibited dietary cohesion in dry seasons (figure 4). However, the social foraging ecologies revealed by the two timeseries also differed in two critical ways. First, in wet seasons, the Artists maintained strong dietary cohesion while the Royals' diets became more individualistic (figure 4). Second, both families established more cohesive dry-season diets, but the plant richness in Artists’ diets expanded whereas the Royals' diets did not (figure 3).

Although our ability to directly compare the two timeseries is limited due to different sampling years and strategies, independent data on the inter-individual relationships, reproductive statuses, movement histories of each family at the time of sampling can provide critical insights into the different patterns of within-group cohesion and differentiation [18,29]. At the time of sampling, the Artists’ core family group comprised the matriarch and her adult offspring while the Royals comprised two high ranking (and older) sisters. In the early stage of the Royals' timeseries, Cleopatra gave birth (13 Oct 2005) and Anastasia was weaning her youngest offspring (age 2–3 years during sampling). In contrast, the youngest Artists’ calves were of more similar ages (greater than 2 years old and not lactationally dependent). As such, the reproductive stages of the two Royals that we tracked were more strongly differentiated than the four Artists in terms of nutritional requirements for offspring and this difference could be reflected in the stronger degree of foraging individuality through this time series. Meanwhile, the timeseries for the subdominant Artists showed stronger seasonal diet switching and greater within-season cohesion than the socially dominant Royals (figure 4; electronic supplementary material, datasets S11–S14), including a strong pattern of dry-season dominance of woody acacias (i.e. *Vachellia* and *Senegalia*; figures 2 and 3; electronic supplementary material, dataset S9). Elephants generally avoid foraging in low-diversity woodlands, especially in dry seasons when the predominant availability of a few woody species can force them to sacrifice foraging efficiency as they must increase time spent searching for rare resources so they can maintain the dietary diversity needed to obtain adequate nutrition and avoid phytochemical toxicity [63]. If the Artists were forced to spend more time foraging in low-diversity woodlands, this could at least partly explain their 2.4-fold greater median daily distance travelled, their diets that were strongly dominated by woody acacias despite a greater variety of rare resources, and their stronger seasonal diet-switching behaviour [36]. By contrast, if different sampling strategies influenced our ability to detect seasonal variation, the Royals' timeseries would likely have had greater power due to more continuous sampling over a time when peak NDVI was higher, and since the samples were approximately 5 years younger and milled into powder prior to storage and analysis [64]. Notwithstanding other environmental influences, the Royals’ higher rank and/or postnatal nutritional requirements may have provided more opportunities or imposed more constraints that promoted use of certain resources rather than engaging in diet-switching behaviors. Better understanding of intrinsic and extrinsic influences on the state and status of individuals in a group that might shape their social foraging behaviors is emerging as a major priority for the field.

### 4.2. Behavioural ecology of diet specialization

We considered alternative predictions about seasonal variation in the contributions of individuals to group foraging through the lenses of Optimal Foraging Theory and the Niche Variation Hypothesis. Evidence suggests that populations of diverse species differ in the degree to which they match the predictions of both theories [1,2], and that large mammalian herbivores have highly plastic diets that may generally be aligned with predictions based on Optimal Foraging [14,35]. Our data matched the expectation that elephant diets are plastic but departed from both sets of predictions as dietary richness either expanded (Artists) or did not reveal a strong correlation (Royals) with increasing NDVI, while familial cohesion levels were relatively strong in dry seasons (figures 3 and 4). Theoretically, individual specialization is a hallmark of intraspecific competition for food, but the Artists generally exhibited cohesion while the Royals became more individualistic with resource abundance (figure 4). The Royals, as the dominant family, were perhaps more consistently able to occupy desirable habitats and avoid extensive daily movements, potentially enabling more individuality and ameliorating a need for cohesive diet switching [36,63]. Further, the Royals had divergent reproductive states, which potentially generated different resource needs. By partitioning diet variation into within- and between-individual components, we revealed variation in the behavioural ecologies of social groups—with their complex bonds, demographic structures and competitive hierarchies—that have the potential to modulate trophic networks in ways that cannot be seen in aggregate data [35,65,66].

### 4.3. Roadmap to integrate DNA and isotopes

Stable isotopes measure proportional C_3_ versus C_4_ plant consumption while DNA metabarcoding provides complementary information on food identity. Our estimates of C_4_-grass DNA and isotope proportions were positively correlated, but varied considerably (figure 3; electronic supplementary material, figures S3–S5). A simplistic interpretation is that DNA-based methods are prone to biases estimating RRA [67,68], but evidence increasingly supports using RRA with well-established protocols [56,57,59]. Another possibility concerns the isotopic diversity of local plants. Through time and space, drought stress produces *δ*^13^C values that are more positive in C_3_ plants and more negative in C_4_ plants (reducing differences between groups). Likewise, C_3_ plants in xeric habitats are consistently more positive than C_3_ plants in riparian zones (again, reducing the difference). Thus, estimates of grass consumption by wide-ranging and long-lived animals may benefit from using different plant tissues as reference points through time and space [69]. A related possibility involves variation in the DNA content of plant tissues. Because chloroplast DNA density is expected to be highest in nitrogen-rich tissues, disparities in % grass DNA could reflect variation in grass contributions to animal protein budgets [19,65]. Taxonomically, the DNA also revealed that elephants eat non-grass families that may include C_4_ or CAM species (e.g. Amaranthaceae, Cleomaceae, Zygophyllaceae; electronic supplementary material, dataset S9). Consumption of some taxa increased with NDVI in similar ways to what was expected for grasses (e.g. C_4_, *Blepharis edulis*, Acanthaceae; C_3_, C_4_, or C_3_–C_4_ intermediate, *Euphorbia* spp., Euphorbiaceae; electronic supplementary material, datasets S10–S13). Prior isotopic estimates of % C_4_ consumption revealed 4-fold differences depending on reference plant material used, and the diversity of plant taxa contributing to elephant diets suggests that grasses may not be the sole determinants of *δ*^13^C [28]. Recognizing that diets vary in multiple dimensions—as opposed to a simple gradient between C_4_ grasses and all other plants—should spur further efforts to integrate genetic and isotopic data in the study of wildlife ecology.

## 5. Conclusion

Social foraging is a widespread phenomenon, and theoretically it confers benefits over solitary foraging by maximizing animals' abilities to locate and monopolize high-quality foods, but a major challenge in behavioural ecology is to assess the costs and benefits of social foraging with knowledge of how individuals use resources [11,13]. Prior studies revealed long-term foraging histories for the elephants of Samburu and Buffalo Springs using stable isotopes [27,28,46], and archived fecal samples from these same animals enabled us to reconstruct high-resolution dietary profiles using DNA. Dietary DNA illuminated a complex pattern of short-term dietary variation that was nested within the broad groups of C_3_ and C_4_ plant resources: individual diets varied in richness and composition as elephants used a diversity of available C_3_ and C_4_ plant species on daily timescales. Since plants of similar isotopic values can represent phylogenetically disparate taxa and divergent nutritional values, dietary stable isotopes and DNA metabarcoding approaches represent synergistic ways of obtaining high-precision dietary timeseries from wildlife [20]. Although our sampling strategy was not designed to elucidate general rules of social foraging, the unprecedented granularity of these two timeseries revealed striking contrasts in dietary patterns within and between well-studied elephants. Access to such timeseries may ultimately help determine social controls on the emergence of diverse foraging cultures, group cohesion, and the individuality of nutrition in wildlife. Theoretically, individual specialization allows groups to enhance their cultural repertoire, but groups that distribute behavioural diversity across individuals can be more susceptible to losses [70]. This double-edged sword could be reflected in the diverse ways that elephant groups from northern Kenya have responded to shifting hotspots of poaching risk and resource availability in recent generations [71].

## Data Availability

R code, DNA barcode reference libraries, and satellite imagery are available at Dryad Digital Repository: https://doi.org/10.5061/dryad.vt4b8gtvs [72]. DNA metabarcoding data are available at NCBI SRA (PRJNA984689). The data are provided in electronic supplementary material [73].
